# *Vital Signs*: State-Level Variation in Nonfatal and Fatal Cardiovascular Events Targeted for Prevention by Million Hearts 2022

**DOI:** 10.15585/mmwr.mm6735a3

**Published:** 2018-09-07

**Authors:** Matthew D. Ritchey, Hilary K. Wall, Pamela L. Owens, Janet S. Wright

**Affiliations:** ^1^Division for Heart Disease and Stroke Prevention, CDC; ^2^Center for Delivery, Organization and Markets, Agency for Healthcare Research and Quality, Rockville, Maryland.

## Abstract

**Introduction:**

Despite its preventability, cardiovascular disease remains a leading cause of morbidity, mortality, and health care costs in the United States. This study describes the burden, in 2016, of nonfatal and fatal cardiovascular events targeted for prevention by Million Hearts 2022, a national initiative working to prevent one million cardiovascular events during 2017–2021.

**Methods:**

Emergency department (ED) visits and hospitalizations were identified using Healthcare Cost and Utilization Project databases, and deaths were identified using National Vital Statistics System data. Age-standardized Million Hearts–preventable event rates and hospitalization costs among adults aged ≥18 years in 2016 are described nationally and across states, as data permit. Expected 2017–2021 event totals and hospitalization costs were estimated assuming 2016 values remain unchanged.

**Results:**

Nationally, in 2016, 2.2 million hospitalizations (850.9 per 100,000 population) resulting in $32.7 billion in costs, and 415,480 deaths (157.4 per 100,000) occurred. Hospitalization and mortality rates were highest among men (989.6 and 172.3 per 100,000, respectively) and non-Hispanic blacks (211.6 per 100,000, mortality only) and increased with age. However, 805,000 hospitalizations and 75,245 deaths occurred among adults aged 18–64 years. State-level variation occurred in rates of ED visits (from 56.4 [Connecticut] to 274.8 per 100,000 [Kentucky]), hospitalizations (484.0 [Wyoming] to 1670.3 per 100,000 [DC]), and mortality (111.2 [Vermont] to 267.3 per 100,000 [Mississippi]). Approximately 16.3 million events and $173.7 billion in hospitalization costs could occur during 2017–2021 without preventive intervention.

**Conclusions and Implications for Public Health Practice:**

Million Hearts–preventable events place a considerable health and economic burden on the United States. With coordinated efforts, many of these events could be prevented in every state to achieve the initiative’s goal.

## Introduction

Heart disease and stroke are largely preventable (*1*–*3*). However, despite decades-long improvement in outcomes, they remain leading causes of morbidity, mortality, and health care costs in the United States (*2*). Moreover, considerable disparities persist and recent evidence suggests that heart disease and stroke event rates are increasing among certain demographic groups, including adults aged 35–64 years (*2*,*4*). In response, CDC and the Centers for Medicare & Medicaid Services launched Million Hearts 2022, a national initiative working to prevent one million heart attacks, strokes, and other acute cardiovascular events during 2017–2021 (*1*,*5*).

Million Hearts 2022, in collaboration with multiple federal, state, and nongovernmental partners, supports the implementation of a selected set of evidence-based public health and clinical strategies aimed at keeping adults healthy and optimizing care to prevent cardiovascular events. This includes using strategies that improve the “ABCS” (aspirin when appropriate, blood pressure control, cholesterol management, and smoking cessation) of cardiovascular care; reducing sodium consumption, tobacco use, and physical inactivity; improving care among persons who have had cardiovascular events*; and addressing known disparities in cardiovascular outcomes^†^ (*6*). Despite their efficacy, implementation of these strategies throughout the country has been inconsistent, which might contribute to the disparities and geographic variation observed in cardiovascular disease (CVD) outcomes (*2*,*4*).

This study describes the distribution, by demographic characteristics and state, of nonfatal (emergency department [ED] visits and hospitalizations) and fatal cardiovascular events that occurred during 2016 and are being targeted for prevention by Million Hearts 2022. Furthermore, it provides a baseline for states by estimating the number of events and hospitalization costs expected to occur if 2016 rates remain unchanged during 2017–2021. These findings can be used by Million Hearts 2022 partners to understand the recent and potential future event burden if no further intervention occurs, and to focus their use of prevention strategies and assess the potential effect on cardiovascular event totals.

## Methods

This study leverages a previously published methodology to identify mutually exclusive nonfatal and fatal cardiovascular events (Million Hearts–preventable events) among adults aged ≥18 years attributed to acute myocardial infarctions, strokes, precursor cardiovascular conditions, and other cardiovascular conditions, by applying specified *International Classification of Diseases, Tenth Revision* (ICD-10) codes within administrative data (Supplementary Table 1, https://stacks.cdc.gov/view/cdc/58170) (*7*).^§^ Where data availability permit, event rates are described at the state and national levels for 2016 (most current available data). At the state level, the Agency for Healthcare Research and Quality’s (AHRQ)^¶^ Healthcare Cost and Utilization Project (HCUP) State Emergency Department Database (SEDD) was used to describe 2016 treat-and-release ED event rates for 34 states and the District of Columbia (DC)** and the HCUP State Inpatient Database (SID) was used to describe 2016 acute, nonfatal hospitalization event rates for 46 states and DC.^††^ Data to generate weighted national estimates for 2016 ED events were unavailable at the time of publication; these data^§§^ will be used for national Million Hearts surveillance as they become available. Weighted national estimates and standard errors were determined for 2016 hospitalization events by using HCUP’s National Inpatient Sample (NIS),^¶¶^ a database developed from data collected via the State Inpatient Database. The National Center for Health Statistics’ National Vital Statistics System*** Mortality Data were used to describe 2016 mortality rates for the nation and all 50 states and DC.

Event rates were stratified by gender and age group (18–44, 45–64, 65–74, and ≥75 years), race and Hispanic origin (mortality only^†††^), event type (acute myocardial infarction, stroke, precursor condition, and other condition), and state.^§§§^ Overall mutually exclusive event rates equal the sum of the treat-and-release ED visits, acute, nonfatal hospitalizations, and deaths and are available for 35 jurisdictions with complete data. Rates were standardized by age to the 2010 U.S. Census. Costs associated with hospitalizations were determined by applying HCUP cost-to-charge ratios^¶¶¶^ to the hospital billing charges provided in the SID (state estimates) and NIS (national estimates) and are presented in 2016 US$; these costs exclude professional (physician) fees. The mean cost per hospitalization was calculated and standardized by age and national event type distribution.**** Age-standardized per capita hospitalization costs, representing the overall cost per adult aged ≥18 years living in the jurisdiction, are presented at the national and state levels.

State-level estimates for the number of Million Hearts–preventable events and hospitalization costs (in 2016 US$) expected to occur during 2017–2021 were calculated in two ways. For states with complete 2016 data (ED, hospitalization, and mortality estimates), the overall age-specific mutually exclusive rates for 2016 were applied to the projected state population estimates^††††^ during 2017–2021 and summed to determine the expected event totals; the 2016 mean state- and age-specific cost per hospitalization was applied to the expected hospitalization event total to estimate expected costs. For states with incomplete 2016 data, it was assumed that the proportional relationship across their ED, hospitalization and mortality rates were the same as the average calculated among states with complete data. If a state was missing 2016 hospitalization data, the national age-specific average cost per hospitalization event was applied to their expected age-specific hospitalization event totals and summed. Expected overall U.S. event totals and hospitalization costs during 2017–2021 equal the sum of the state-level estimates.^§§§§^

## Results

Nationally, in 2016, over 2.2 million Million Hearts–preventable hospitalizations and 415,480 deaths occurred (Table 1). The hospitalizations resulted in an estimated $32.7 billion in costs. For both event types, the burden was higher among men than among women (age-standardized hospitalization rates of 989.6 and 725.1 per 100,000 population, respectively, and mortality rates of 172.3 and 143.0 per 100,000, respectively) and increased with age. However, an estimated 805,000 hospitalizations and 75,245 deaths occurred among adults aged 18–64 years. Among all racial/ethnic groups, the highest mortality rates were in non-Hispanic blacks (211.6 per 100,000). Acute myocardial infarctions and strokes accounted for approximately half (47%) of hospitalizations (rates of 204.5 and 199.1 per 100,000, respectively) and approximately two thirds (61%) of deaths (42.2 and 53.7 per 100,000, respectively). “Other” cardiovascular events, which include those related to heart failure, contributed to 46% of hospitalizations and 38% of deaths (rates = 394.6 and 59.8 per 100,000, respectively).

**TABLE 1:** National Million Hearts–preventable hospitalization and mortality rates (per 100,000 population) and hospitalization costs among adults aged ≥18 years, by age group, gender, race-ethnicity* and event type, 2016

Event	No., thousands (SE^†^)	Cost^§^ (SE^†^), in US$ billions	Crude rate (SE^†^)	Age-standardized rate^¶^ (SE^†^)
**Acute hospitalizations**				
**Total**	**2,238.3 (24.6)**	**32.7 (0.29)**	**897.2 (9.9)**	**850.9 (5.8)**
**Men (total)**	1,180.1 (13.6)	18.6 (0.18)	971.5 (11.2)	989.6 (7.1)
**Age group (yrs), men**				
18–44	73.0 (1.2)	1.3 (0.03)	124.6 (2.0)	—
45–64	426.0 (5.6)	7.4 (0.09)	1,036.4 (13.6)	—
65–74	286.1 (3.7)	4.8 (0.05)	2,136.5 (27.3)	—
≥75	395.0 (4.9)	5.1 (0.04)	4,700.9 (58.2)	—
**Women (total)**	1,057.2 (11.4)	14.1 (0.12)	825.8 (8.9)	725.1 (5.1)
**Age group (yrs), women**				
18–44	46.9 (0.9)	0.8 (0.02)	81.6 (1.5)	—
45–64	258.7 (3.5)	4.1 (0.05)	599.6 (8.0)	—
65–74	231.1 (2.8)	3.3 (0.03)	1,516.6 (18.4)	—
≥75	520.5 (6.0)	5.9 (0.04)	4,262.2 (48.9)	—
**Event type**				
AMI	536.3 (8.7)	11.6 (0.09)	215.0 (3.5)	204.5 (2.0)
Stroke	524.3 (7.5)	8.4 (0.12)	210.2 (3.0)	199.1 (1.8)
Precursor**	138.4 (2.1)	1.1 (0.01)	55.5 (0.8)	52.7 (0.5)
Other^††^	1,039.3 (11.0)	11.6 (0.14)	416.6 (4.4)	394.6 (2.9)
**Deaths**				
Total	415.5	NA	166.5	157.4
**Men (total)**	199.4	NA	164.1	172.3
**Age group (yrs), men**				
18–44	5.2	NA	8.9	—
45–64	44.2	NA	107.6	—
65–74	42.0	NA	313.5	—
≥75	107.9	NA	1,284.4	—
**Women (total)**	216.1	NA	168.8	143.0
**Age group (yrs), women**				
18–44	2.7	NA	4.7	—
45–64	23.1	NA	53.5	—
65–74	28.3	NA	185.4	—
≥75	162.1	NA	1,327.2	—
Race/Ethnicity*				
White, non-Hispanic	320.2	NA	197.9	160.2
Black, non-Hispanic	52.2	NA	170.6	211.6
Hispanic^§§^	25.4	NA	66.4	114.9
Other, non-Hispanic	12.6	NA	75.2	97.1
Asian/PI	10.6	NA	71.5	92.3
AI/AN	2.0	NA	103.6	132.9
**Event type**				
AMI	111.7	NA	44.8	42.2
Stroke	141.8	NA	56.9	53.7
Precursor**	4.4	NA	1.7	1.7
Other^††^	157.5	NA	63.1	59.8

**Sources:** Agency for Healthcare Research and Quality’s Healthcare Cost and Utilization Project (HCUP) National Inpatient Sample (NIS); National Center for Health Statistics’ National Vital Statistics System Mortality Data.

**Abbreviations:** AI/AN = American Indian/Alaskan Native; AMI = acute myocardial infarction; Asian/PI = Asian/Pacific Islander; NA = not applicable; SE = standard error.

* Race/ethnicity information was consistently available nationally for only mortality data. During 1999–2011, the sensitivity for identifying the correct race and ethnicity on death certificates was 99.2% (non-Hispanic whites), 98.1% (non-Hispanic blacks), 91.3% (Hispanics), 93.5% (Asian/PI), and 73.3% (AI/AN) (Arias E, Heron M, Hakes JK. The validity of race and Hispanic-origin reporting on death certificates in the United States: An update. National Center for Health Statistics. Vital Health Stat 2 2016;172:1–21. https://www.cdc.gov/nchs/data/series/sr_02/sr02_172.pdf).

^†^ Standard errors are provided only for acute hospitalization estimates as they are determined using a sample of hospitalizations (NIS) obtained from the HCUP State Inpatient Databases. No sampling error is produced when using mortality data from the National Vital Statistic System.

^§^ Described by applying HCUP cost-to-charge ratios to the charges the hospitals billed for the entire hospital stay; these costs exclude professional (physician) fees (https://www.hcup-us.ahrq.gov/db/state/costtocharge.jsp). The age- and event type-standardized mean cost per event in the United States was $16,274 per hospitalization and the age-standardized per-capita cost was $125 per U.S. adult.

^¶^ Standardized by age to the 2010 U.S. Census population.

** Includes stable angina pectoris, transient ischemic attack, and other acute and subacute ischemic heart disease.

^††^ Includes heart failure, abdominal aortic aneurysm, atheroembolism (hospitalizations only), atherosclerosis and peripheral artery disease (deaths only), hypertension without heart failure, and cardiac arrest that had another Million Hearts–preventable event type coded as a secondary diagnosis or contributing cause of death.

^§§^ Persons with unspecified ethnicity were considered to be non-Hispanic (approximately 0.3% of deaths).

Age-standardized event rates per 100,000 population varied considerably across states with available data, including for treat-and-release ED visits (34 states and DC; range = 56.4 [Connecticut] to 274.8 [Kentucky]), acute hospitalizations (46 states and DC; range = 484.0 [Wyoming] to 1670.3 [DC]), and deaths (50 states and DC; range = 111.2 [Vermont] to 267.3 [Mississippi]) (Table 2) (Supplementary Figure 1, https://stacks.cdc.gov/view/cdc/58168). Among the 35 jurisdictions with complete overall data, the three with the lowest overall mutually exclusive event rates were Utah (805.7), Wyoming (828.9), and Vermont (840.6) and those with the highest rates were DC (2,048.2), Tennessee (1,551.6), and Kentucky (1,510.3) (Figure).

**TABLE 2:** Age-standardized Million Hearts–preventable emergency department, hospitalization, mortality rates (per 100,000 population), hospitalization costs, and overall event totals among adults aged ≥18 years, by state*— United States, 2016

State	Treat-and-release ED visit rate^†^	Acute hospitalizations				Mortality rate^†^	Overall event total (thousands)^††^
		Rate^†^	Cost, in US$ (2016) billions	Mean cost (US$) per event^§,¶^	Per-capita costs (US$)^§,^**		
Alabama	—^§§^	—^§§^	—^§§^	—^§§^	—^§§^	206.1	—^§§^
Alaska	—^§§^	593.0	0.07	24,017	149	116.9	—^§§^
Arizona	132.8	666.7	0.56	14,935	97	114.4	53.9
Arkansas	192.5	914.2	0.24	11,307	95	260.0	34.2
California	154.7	698.3	4.21	23,092	143	146.4	294.9
Colorado	—^§§^	555.1	0.38	18,479	91	123.9	—***
Connecticut	56.4	773.5	0.42	19,256	133	120.5	30.1
Delaware	—^§§^	—^§§^	—^§§^	—^§§^	—^§§^	131.7	—^§§^
District of Columbia^¶¶^	202.0	1,670.3	0.13	20,600	294	175.9	9.2
Florida	113.4	916.0	2.30	13,907	116	134.3	235.1
Georgia	233.5	928.6	0.89	14,171	117	188.9	101.0
Hawaii	149.8	755.7	0.17	18,573	141	126.3	12.7
Idaho	—^§§^	—^§§^	—^§§^	—^§§^	—^§§^	156.3	—^§§^
Illinois	140.0	861.7	1.30	17,130	127	173.0	120.3
Indiana	200.8	960.1	0.68	14,122	128	177.3	71.3
Iowa	199.2	670.7	0.24	15,442	90	138.0	27.8
Kansas	184.5	754.3	0.22	13,507	96	168.1	26.1
Kentucky	274.8	1,025.2	0.56	16,591	153	210.3	55.2
Louisiana	—^§§^	1,097.0	0.47	12,622	130	213.4	—^§§^
Maine	237.6	784.3	0.17	19,136	134	136.0	15.3
Maryland	165.7	787.2	0.47	13,762	97	153.7	53.1
Massachusetts	64.9***	839.1	0.78	20,720	135	129.1	60.4
Michigan	—^§§^	1,013.1	1.11	14,937	131	176.3	—^§§^
Minnesota	127.4	659.7	0.52	20,228	114	113.1	40.9
Mississippi	—^§§^	1,040.0	0.29	12,216	122	267.3	—^§§^
Missouri	179.9	999.5	0.71	14,813	138	202.1	71.3
Montana	165.9	546.9	0.08	13,744	81	136.6	8.0
Nebraska	142.9	645.0	0.16	17,866	104	141.7	14.4
Nevada	169.3	804.1	0.27	14,105	115	134.0	25.4
New Hampshire	—^§§^	—^§§^	—^§§^	—^§§^	—^§§^	126.9	—^§§^
New Jersey	129.8	839.5	0.99	17,308	131	138.7	83.6
New Mexico	—^§§^	528.9	0.13	15,568	76	133.1	—^§§^
New York	91.1	803.9	2.28	19,676	138	134.8	169.9
North Carolina	195.9	947.6	1.00	14,132	121	159.7	107.8
North Dakota	162.8	912.4	0.09	18,224	157	134.8	7.3
Ohio	190.8	996.8	1.33	14,866	134	176.4	136.6
Oklahoma	—^§§^	884.8	0.35	13,539	112	197.4	—^§§^
Oregon	—^§§^	675.4	0.39	18,989	110	138.6	—^§§^
Pennsylvania	—^§§^	987.3	1.55	15,986	133	162.5	—^§§^
Rhode Island	148.6	932.8	0.12	15,480	129	131.2	11.5
South Carolina	235.8	921.8	0.49	14,125	118	169.1	55.4
South Dakota	167.3	715.5	0.08	15,594	104	174.9	7.8
Tennessee	236.6	1,121.0	0.71	12,342	130	194.0	85.0
Texas	201.7	893.7	2.48	15,654	129	168.9	239.1
Utah	116.6	537.8	0.17	19,859	90	151.3	14.4
Vermont	157.9	571.5	0.05	17,876	90	111.2	4.9
Virginia	—^§§^	866.4	0.77	15,727	115	154.6	—^§§^
Washington	—^§§^	713.0	0.72	20,661	125	127.4	—^§§^
West Virginia	—^§§^	1,030.9	0.25	13,416	145	172.8	—^§§^
Wisconsin	145.7	730.7	0.55	17,107	111	148.6	51.2
Wyoming	194.9	484.0	0.04	15,977	76	150.0	3.8

**Sources:** Agency for Healthcare Research and Quality’s Healthcare Cost and Utilization Project (HCUP), State Emergency Department Databases and State Inpatient Databases; National Center for Health Statistics’ National Vital Statistics System Mortality Data.

**Abbreviation:** ED = emergency department.

* Calculated only for states where data were made available.

^†^ Standardized by age to the 2010 U.S. Census population.

^§^ Described by applying HCUP cost-to-charge ratios to the charges the hospitals billed for the entire hospital stay in each state; these costs exclude professional (physician) fees.

^¶^ Standardized by age to the 2010 U.S. Census population and by event type to the national distribution of events (acute myocardial infarctions, strokes, precursor events, and other cardiovascular events) observed during 2016.

** Represents the overall cost per adult aged ≥18 years living in the jurisdiction, standardized by age to the 2010 U.S. Census population.

^††^ Represents the sum of number of treat-and-release ED visits, acute, nonfatal hospitalizations, and deaths that occurred in that jurisdiction.

^§§^ Data were not collected or were not available for analysis (treat-and-release ED data for Mississippi and Oregon are regularly collected, but 2016 data were not available at time of this report).

^¶¶^ The ED and hospitalization event and cost values are likely overestimates for this location because these events are attributed to where the event was treated and not the residence of the patient.

*** Transfers to other acute care hospitals are not identified in discharge disposition codes, so they could not be excluded from the analysis and the rate may be slightly inflated.

**FIGURE:**
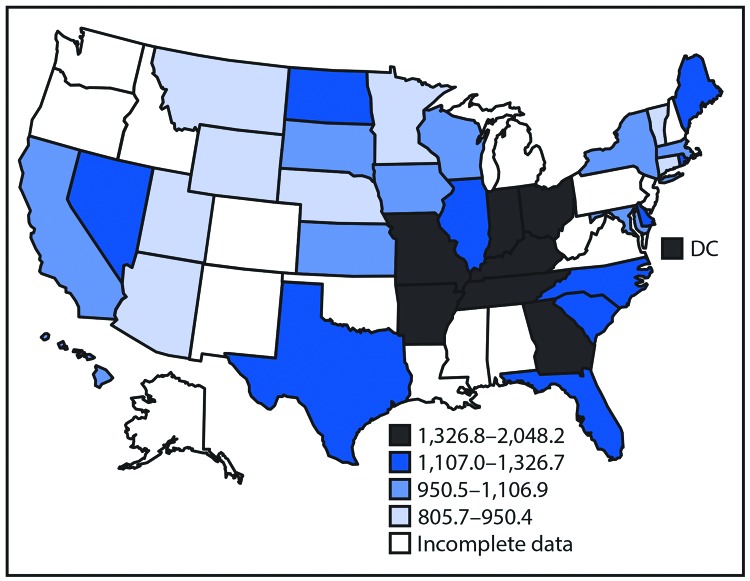
Age-standardized overall Million Hearts–preventable event* rates among adults aged ≥18 years, by U.S. state,^^†^^ 2016 **Sources:** Agency for Healthcare Research and Quality’s Healthcare Cost and Utilization Project State Emergency Department Databases and State Inpatient Databases; National Center for Health Statistics’ National Vital Statistics System Mortality Data. * Includes mutually exclusive nonfatal treat-and-release emergency department visits, nonfatal acute hospitalizations and deaths attributed to acute myocardial infarctions, strokes, precursor cardiovascular conditions (e.g., stable angina pectoris), and other cardiovascular conditions (e.g., heart failure). ^^†^^ Complete data are available for 34 states and the District of Columbia (DC). Supplementary Figure 1 shows age-standardized rates of treat-and-release emergency department visits for 34 states and DC, hospitalizations for 46 states and DC, and mortality for 50 states and DC (https://stacks.cdc.gov/view/cdc/58168).

In 2016, the age-standardized per-capita hospitalization cost was $125 in the United States, and ranged across states with available data from $76, in New Mexico and Wyoming, to $294, in DC (Table 2). The age- and event type-standardized mean cost per hospitalization was $16,274 nationally and ranged from $11,307 in Arkansas to $24,017 in Alaska. If the 2016 overall mutually exclusive event rates were to remain constant during 2017–2021, an estimated 16.3 million events are expected to occur, including 2.2 million ED visits, 11.8 million hospitalizations, and 2.2 million deaths (Table 3) (Supplementary Table 2, https://stacks.cdc.gov/view/cdc/58171); if mean hospitalization costs per event remained constant, an estimated $173.7 billion in costs would be expected to occur. Preventing one million events during 2017–2021 would result in approximately a 6.1% reduction in expected event totals and associated costs.

**TABLE 3:** Expected number of Million Hearts–preventable events and hospitalization costs among adults aged ≥18 years during 2017–2021, nationally and by state — United States*

State	Expected event totals, in thousands				Expected hospitalization costs, in US$ (2016) billions
	Treat-and-release ED visits	Acute hospitalizations	Deaths	Total mutually exclusive events	
United States	2,231.3	11,843.8	2,214.0	16,289.1	173.7
Alabama	48.4^†^	255.5^§^	44.1	347.9^¶^	3.8^§^
Alaska	2.8^†^	14.7	2.6	20.1^¶^	0.4
Arizona	39.9	200.9	35.5	276.3	2.9
Arkansas	24.7	118.1	34.5	177.3	1.2
California	241.4	1,088.6	229.0	1,558.9	22.3
Colorado	23.5^†^	122.1	26.5	172.0^¶^	2.0
Connecticut	9.6	131.0	21.1	161.7	2.2
Delaware	6.2^†^	32.9^§^	6.1	45.3^¶^	0.5^§^
District of Columbia	4.9	40.2	4.0	49.1	0.7
Florida	113.9	945.2	147.7	1,206.8	11.8
Georgia	94.2	374.7	74.6	543.5	4.8
Hawaii	9.7	50.4	8.9	69.0	0.9
Idaho	11.4^†^	60.8^§^	11.7	83.8^¶^	0.9^§^
Illinois	74.1	458.2	93.0	625.4	6.7
Indiana	56.4	270.9	50.7	377.9	3.6
Iowa	28.7	96.0	20.7	145.4	1.3
Kansas	22.7	92.4	21.2	136.3	1.2
Kentucky	52.8	197.9	40.9	291.6	2.9
Louisiana	39.0^†^	201.7	38.5	279.3^¶^	2.4
Maine	17.0	55.3	9.9	82.3	0.9
Maryland	41.7	199.6	38.9	280.2	2.5
Massachusetts	19.6	254.8	39.5	314.0	4.1
Michigan	84.7^†^	457.2	81.6	623.5^¶^	5.9
Minnesota	31.1	159.4	28.0	218.4	2.7
Mississippi	25.8^†^	126.5	32.7	185.0^¶^	1.5
Missouri	48.5	270.2	56.5	375.3	3.7
Montana	8.7	27.9	7.2	43.8	0.4
Nebraska	11.7	52.6	11.9	76.2	0.8
Nevada	19.5	93.3	15.2	128.0	1.3
New Hampshire	7.6^†^	40.9^§^	8.1	56.6^¶^	0.6^§^
New Jersey	50.1	328.9	55.2	434.2	5.1
New Mexico	9.7^†^	49.2	12.6	71.5^¶^	0.7
New York	77.8	692.1	119.3	889.2	11.8
North Carolina	87.8	425.9	73.1	586.7	5.4
North Dakota	5.4	30.2	4.5	40.1	0.5
Ohio	99.4	524.3	95.3	718.9	7.0
Oklahoma	28.2^†^	143.6	32.4	204.1^¶^	1.8
Oregon	24.3^†^	128.7	27.3	180.3^¶^	2.1
Pennsylvania	110.7^†^	606.3	103.7	820.6^¶^	8.0
Rhode Island	7.2	45.6	6.6	59.3	0.6
South Carolina	52.7	207.3	38.9	298.8	2.6
South Dakota	6.6	28.2	7.1	41.9	0.4
Tennessee	68.1	327.2	57.3	452.6	3.8
Texas	207.2	917.2	167.6	1,291.9	13.4
Utah	11.6	53.5	14.6	79.8	0.9
Vermont	4.9	17.5	3.4	25.9	0.3
Virginia	57.2^†^	306.6	54.4	418.2^¶^	4.1
Washington	41.4^†^	223.8	40.0	305.2^¶^	3.9
West Virginia	17.4^†^	93.9	16.3	127.7^¶^	1.3
Wisconsin	38.7	192.2	40.0	270.9	2.9
Wyoming	4.8	11.9	3.7	20.4	0.2

**Sources:** Agency for Healthcare Research and Quality’s Healthcare Cost and Utilization Project State Emergency Department Databases (from all states except Alabama, Alaska, Colorado, Delaware, Idaho, Louisiana, Michigan, Mississippi, New Hampshire, New Mexico, Oklahoma, Oregon, Pennsylvania, Virginia, Washington and West Virginia; ED data for Mississippi and Oregon are regularly collected, but 2016 data were not available at time of this report) and State Inpatient Databases (from all states except Alabama, Delaware, Idaho, and New Hampshire); National Center for Health Statistics’ National Vital Statistics System Mortality Data.

**Abbreviation:** ED = emergency department.

* State-level estimates for the number of Million Hearts–preventable events (those targeted for prevention by the initiative) and hospitalization costs expected to occur during 2017–2021 were calculated in two ways. For states with complete 2016 data (ED, hospitalization, and mortality estimates), the overall age-specific mutually exclusive rates for 2016 were applied to the projected state population estimates during 2017–2021 and summed to determine the expected event totals; the 2016 mean state- and age-specific cost per hospitalization was applied to the expected hospitalization event total to estimate expected costs. For states with incomplete 2016 data, it was assumed that the proportional relationship across their ED, hospitalization, and mortality rates was the same as the average calculated among states with complete data. If a state was missing 2016 hospitalization data, the national age-specific average cost per hospitalization event was applied to their expected age-specific hospitalization event totals and summed. Expected overall U.S. event totals and costs during 2017–2021 equals the sum of the state-level estimates; this method differs from the method being used to officially track these estimates at the national level.

^†^ ED rate was missing, therefore, an estimate was used.

^§^ Hospitalization rate and cost per hospitalization information were missing, therefore, estimates were used.

^¶^ Calculated by using estimated ED visit rates and, where applicable, estimated hospitalization rates and summing those resultant event counts with the known death counts.

## Conclusion and Comment

The subset of cardiovascular events^¶¶¶¶^ targeted for prevention by Million Hearts 2022 places a considerable burden on the health and economic well-being of Americans (*2*,*7*). Despite these events being highly preventable (*3*), they accounted for approximately 2 million hospitalizations and 400,000 deaths in 2016. Furthermore, without a more concerted effort to improve CVD risk factors, an estimated 16.3 million nonfatal and fatal cardiovascular events and $173.7 billion in hospitalization costs are expected to occur during 2017–2021.

Considerable disparities in event rates were evident. Nationally, non-Hispanic blacks continue to experience the highest CVD mortality rates (32% higher than those in non-Hispanic whites). This disparity is due, in part, to the high prevalence of uncontrolled blood pressure among blacks (*6*), placing them at higher risk than other racial/ethnic groups for acute myocardial infarction, stroke, and other CVD conditions, including heart failure (*2*,*8*). Additionally, despite the considerable increase in risk for a cardiovascular event with increasing age, in 2016, over 800,000 combined hospitalizations and deaths occurred among adults aged <65 years (approximately one in three events). Other studies have shown that decades-long improvement in heart disease and stroke mortality have stalled (*9*,*10*) and that younger populations, especially those aged 35–64 years, are experiencing worse outcomes across the country (*4*,*9*). In 2016, Million Hearts-preventable event rates among persons aged 35–64 years varied considerably by demographic characteristics and U.S. state (Supplementary Table 3, https://stacks.cdc.gov/view/cdc/58172); among the overall 16.3 million events expected to occur during 2017–2021 if no additional action is taken, 5.0 million (30.9%) are expected to occur among this age group (Supplementary Figure 2, https://stacks.cdc.gov/view/cdc/58169). Therefore, implementation of strategies that focus on the prevention, early diagnosis, and effective management of CVD risk factors among younger adults is needed to prevent events in both the short- and long-term.

This is one of the first studies to demonstrate striking state-level variation in nonfatal cardiovascular event rates and hospitalization costs using data collected among adults of all ages and across all payer types, including the uninsured. Whereas the burden of state-level mortality was higher in the southeastern United States, which aligns with the findings from previous studies (*2*), rates for ED visits and hospitalizations were higher in both this region and elsewhere, including many Midwestern states. The overall state variation in nonfatal event rates and associated hospitalization costs is likely driven by both geographic differences in disease prevalence and severity, and differences in care delivery and public health quality (*2**,**11*). Additional focus on improving the environments in which persons live, work, and play (e.g., built environment modifications to promote increased physical activity) (*12*), leveraging community resources to aid in CVD risk factor management (e.g., referral to nutritional and fitness counseling groups) (*13*), providing effective outpatient care (e.g., use of team-based care for hypertension and cholesterol management) (*14*), and improving the care received after a cardiovascular event (e.g., systematic referral to cardiac rehabilitation services for those with eligible diagnoses) (*15*) might reduce the need for and the expense of many of these acute care services.

This study uses the best available data to describe the burden of Million Hearts–preventable events at the national and state levels. However, the findings in this report are subject to at least six limitations. First, not all jurisdictions provided ED and/or hospitalization data; efforts to impute missing values might produce inaccurate estimates. Second, nonfatal events are attributed to treatment location and not patient residence, therefore jurisdictions in close proximity to large population centers in neighboring states (e.g., DC) could have overestimated or underestimated rates. Third, whereas the methodology used attempts to identify mutually exclusive events, there is potential for over‐ or undercounting events. Fourth, cardiovascular events that do not result in ED or hospital use or death are not counted. Fifth, because administrative data are being used, differences in use of health care (e.g., changes in how events are medically managed) or coding practices (e.g., changes in how nonfatal events are billed) might affect the event rates and costs presented in this study rather than changes in disease burden. However, this study attempts to address differences in practice patterns by excluding elective hospitalizations and including certain ED events (e.g., heart failure-related visits). Finally, the hospitalization cost estimates are likely conservative, as they do not include professional (physician) fees, and costs were not available for treat-and-release ED visits.

Each state would need to realize an approximate 6% decrease in its expected event totals during 2017–2021 to collectively prevent one million events at the national level. This is feasible if clinical and public health partners in every state mobilize and strengthen their focus on implementing the prevention strategies outlined by Million Hearts 2022 (https://millionhearts.hhs.gov/files/MH-Framework.pdf) to achieve 80% or greater performance on the ABCS and at least a 20% reduction in physical inactivity, tobacco use prevalence, and sodium consumption (*16*).

**Summary**

**What is already known about this topic?**

The health and economic burden of cardiovascular disease is considerable. Million Hearts 2022 supports use of evidence-based clinical and community strategies to prevent one million cardiovascular events during 2017–2021.

**What is added by this report?**

Nationally, in 2016, 2.2 million hospitalizations, costing $32.7 billion, and 415,480 deaths occurred that are being targeted for prevention by Million Hearts 2022, with disparities across demographic characteristics and states. Approximately 16.3 million events could occur during 2017–2021 without preventive intervention.

**What are the implications for public health practice?**

Achieving the Million Hearts 2022 goal likely requires states to focus on using prevention strategies that best meet the cardiovascular health needs of the persons they serve.

## Footnotes

* Includes use of cardiac rehabilitation among persons who have had a qualifying cardiac event or procedure and improving awareness of the potential negative health sequelae of and avoiding exposure to poor air quality among persons who have had a previous cardiovascular event.

† Includes improving hypertension control among blacks/African Americans and improving prevention and management of CVD risk factors among adults aged 35–64 years and persons with mental and/or substance use disorders who use tobacco.

§ Million Hearts–preventable ED visits and hospitalizations were defined as events where patients had one of the specified ICD-10-CM codes listed as their first-listed/principal diagnosis (national implementation of ICD-10-CM codes occurred in October 2015); Million Hearts–related deaths were events with a specified ICD-10 code listed as the underlying cause of death (national implementation of ICD-10 mortality codes occurred in 1999). Precursor cardiovascular conditions include stable angina pectoris, transient ischemic attack, and other acute and subacute ischemic heart diseases. Other cardiovascular conditions include heart failure, abdominal aortic aneurysm, atheroembolism (hospitalizations only), atherosclerosis and peripheral artery disease (deaths only), hypertension without heart failure, and cardiac arrest that had another Million Hearts–preventable event type coded as a secondary diagnosis or contributing cause of death.

¶ AHRQ is a Million Hearts 2022 partner and supported the release of this report. Information about AHRQ’s support of Million Hearts 2022 through their EvidenceNow initiative can be found at https://www.ahrq.gov/sites/default/files/wysiwyg/evidencenow/about/evidence-now-fact-sheet.pdf.

** The SEDD (https://www.hcup-us.ahrq.gov/seddoverview.jsp) is an encounter-based, all-payer (including the uninsured) administrative database capturing claims for 78% of all ED visits that do not result in hospitalization at U.S. community, non-rehabilitation, acute care hospitals. In 2016, it included data from all states except Alabama, Alaska, Colorado, Delaware, Idaho, Louisiana, Michigan, Mississippi, New Hampshire, New Mexico, Oklahoma, Oregon, Pennsylvania, Virginia, Washington, and West Virginia; data for Mississippi and Oregon are regularly collected, but 2016 data were not available at the time of this report. Treat-and-release ED event rates represents ED visits that did not result in a hospitalization, and exclude visits where the patient died in the ED, was transferred to another hospital, or was admitted to the same hospital.

†† The SID (https://www.hcup-us.ahrq.gov/sidoverview.jsp) is an encounter-based, all-payer (including the uninsured) administrative database capturing claims for 97% of all hospitalizations at U.S. community, nonrehabilitation, acute care hospitals. In 2016, it included data from all states except Alabama, Delaware, Idaho, and New Hampshire. To identify acute, nonfatal hospitalizations, hospitalizations that were reported as elective or where the patient died in the hospital or was transferred to another hospital were excluded.

§§ For national Million Hearts surveillance weighted national ED visit rates are determined using HCUP’s Nationwide Emergency Department Sample (NEDS) (https://www.hcup-us.ahrq.gov/nedsoverview.jsp), which contains data from 30.5 million ED visits from about 1,000 hospitals sampled to approximate a 20% stratified sample of U.S. hospital-based emergency departments. Weighted, there are approximately 143 million ED visits annually.

¶¶ The NIS (https://www.hcup-us.ahrq.gov/nisoverview.jsp) approximates a 20% stratified sample of discharges from over 4,500 U.S. community hospitals. Weighted, there are approximately 36 million discharges annually.

*** https://www.cdc.gov/nchs/nvss/index.htm.

††† Race/ethnicity is consistently reported in the SEDD and SID for only 32 states and was therefore not included in the presentation of ED and hospitalization event rates.

§§§ ED and hospitalization events were assigned to the state the patient was treated in. Death events were assigned to the residence the individual lived in.

¶¶¶ Charge information was available for ED and hospitalization events; however, charge amounts represent only what was billed for services and do not reflect how much the services actually cost the hospital to provide or the amount received in payment. Use of HCUP Cost-to-Charge Ratio Files (CCR) (HCUP. 2016. Agency for Healthcare Research and Quality, Rockville, MD. https://www.hcup-us.ahrq.gov/db/state/costtocharge.jsp) allowed for the conversion from charges to costs, but CCRs are only available for hospitalization charges and not for ED charges.

**** Standardized to the 2010 decennial U.S. Census population and to the national distribution of events (percentage of events that were acute myocardial infarctions, strokes, precursor events, or other events) observed during 2016.

†††† State-level population projections were based on the linear extrapolation of 2016 July 1^st^ Census estimates and 2020 population projections provided by the University of Virginia’s Weldon Cooper Center for Public Service (Available at https://demographics.coopercenter.org/national-population-projections and accessed on May 1, 2018).

§§§§ The method used here to describe the number of events and total hospitalization cost expected at the national level differs from the method being used to officially track these estimates at the national level; that method uses estimates generated from using the NEDS, NIS, and NVSS (https://www.ahajournals.org/doi/10.1161/JAHA.117.006021).

¶¶¶¶ Overall, CVD accounts for approximately 800,000 deaths and an estimated $300 billion in direct medical expenses and lost productivity annually (2). The subset of events targeted for prevention by Million Hearts 2022 activities was identified based on multiple factors described by Ritchey et al (https://www.ahajournals.org/doi/10.1161/JAHA.117.006021) with the most important factor being the preventability of the event by current or planned Million Hearts 2022 efforts.
